# Combination of High Zn Density and Low Phytic Acid for Improving Zn Bioavailability in Rice (*Oryza stavia* L.) Grain

**DOI:** 10.1186/s12284-021-00465-0

**Published:** 2021-02-27

**Authors:** Yin Wang, Yusha Meng, Yanping Ma, Lei Liu, Dianxing Wu, Xiaoli Shu, Liqing Pan, Qixian Lai

**Affiliations:** 1 Institute of Rural Development, Zhejiang Academy of Agricultural Sciences, Hangzhou, 310021 China; 2 Key Laboratory of Creative Agriculture, Ministry of Agriculture, Hangzhou, 310021 China; 3 State Key Laboratory of Rice Biology, Institute of Nuclear Agriculture Sciences, Zhejiang University, Hangzhou, 310029 China; 4 Yuyao County Agricultural Techniques Promotion and Service Station, Yuyao Agricultural and Rural Bureau, Ningbo, 315400 China

**Keywords:** Zinc (Zn), Bioavailability, Rice, Cross-breeding, Biofortification

## Abstract

**Background:**

Zn deficiency is one of the leading public health problems in the world. Staple food crop, such as rice, cannot provide enough Zn to meet the daily dietary requirement because Zn in grain would chelate with phytic acid, which resulted in low Zn bioavailability. Breeding new rice varieties with high Zn bioavailability will be an effective, economic and sustainable strategy to alleviate human Zn deficiency.

**Results:**

The high Zn density mutant *LLZ* was crossed with the low phytic acid mutant *Os-lpa-XS110–1*, and the contents of Zn and phytic acid in the brown rice were determined for the resulting progenies grown at different sites. Among the hybrid progenies, the double mutant always displayed significantly higher Zn content and lower phytic acid content in grain, leading to the lowest molar ratio of phytic acid to Zn under all environments. As assessed by in vitro digestion/Caco-2 cell model, the double mutant contained the relatively high content of bioavailable Zn in brown rice.

**Conclusions:**

Our findings suggested pyramiding breeding by a combination of high Zn density and low phytic acid is a practical and useful approach to improve Zn bioavailability in rice grain.

**Supplementary Information:**

The online version contains supplementary material available at 10.1186/s12284-021-00465-0.

## Background

Zinc (Zn) is one of the essential micronutrients required for various biochemical processes and physiological functions in organisms. It could not only participate in enzyme activation, but also play an important role in the metabolism of carbohydrates, lipids, proteins, and nucleic acids (Brocard and Dreno 2011; Kelleher et al. 2011). Severe Zn deficiency in the human body causes a series of metabolic disorders, and even death (Mayer et al. 2008). The World Health Organization estimated that more than 2 billion people worldwide have the problem of Zn deficiency, and the almost half of children and women in low- and middle-income countries suffer from serious Zn deficiency (Caulfield and Black 2004; Wessells and Brown 2012). The Zn deficiency-induced malnutrition constitutes a major public health problem in the world.

Zn deficiency is primarily due to the insufficient intake of bioavailable Zn from foods (Lazarte et al. 2016). Rice is a major diet component for more than half of the world’s population, especially for those residents in southern and eastern Asia. Unfortunately, the current main rice varieties cannot provide enough Zn to meet the daily dietary requirement of Zn due to the low Zn bioavailability (Du et al. 2010; Jou et al. 2012; La Frano et al. 2014). Zn bioavailability is the amount of the absorbed Zn in the blood system that is accessible for utilization in normal physiological functions (La Frano et al. 2014). Zn bioavailability of rice grain is influenced by grain Zn content, and Zn enrichment in rice grain significantly increased the amount of bioavailable Zn (Jou et al. 2012; Sreenivasulu et al. 2008; Wei et al. 2012a, b). In resent year, various strategies have been proposed to improve grain Zn bioavailability by increasing grain Zn content (Jeng et al. 2012; Johnson et al. 2011; Lee et al. 2011; Phattarakul et al. 2012; Wang et al. 2017; Wei et al. 2012a, b). However, several antinutrient compositions, such as phytic acid, could reduce Zn bioavailability by chelating Zn to form indigestible complexes in the human body (Jou et al. 2012; Lonnerdal et al. 2011; Sreenivasulu et al. 2008). Thus, enhancing Zn content synchronizes with decreasing phytic acid might be a valuable way to improve Zn bioavailability in rice.

In this study, a hybrid breeding approach was made to improve Zn bioavailability in rice grain. Two previously developed rice mutants, high Zn density mutant *Lilizhi* (*LLZ*) and low phytic acid mutant *Os-lpa-XS110–1*, were selected as parents to generate a homozygous double mutant that exhibited a phenotype of high Zn content with low phytic acid content in grain. Through assessing the Zn uptake by using an in vitro digestion/Caco-2 cell model, the cross-breeding of two mutants was investigated regarding its impact on the Zn bioavailability of the resulting progenies.

## Materials and Methods

### Chemicals and Reagents

Porcine pepsin, pancreatin and bile salts were purchased from Sigma-Aldrich (St Louis, USA). Dulbecco’s modified Eagle medium (DMEM with glucose 4.5 g/L), fetal bovine serum (FBS), L-glutamine and 4-(2-hydroxyethyl)-1-piperazineethanesulfonic acid (HEPES) were purchased from Gibco Life Technologies (Grand Island, USA). Hanks’ balanced salt solution (HBSS), trypsin-EDTA, nonessential amino acids, penicillin, and streptomycin were purchased from Wisent Life Technologies (Quebec, Canada). All other chemicals were obtained from Sangon Biotech Corporation (Shanghai, China).

### Plant Materials and Growth Conditions

Two rice mutant lines and their wild type parents were used in this study. The mutant *LLZ* was derived from *Oryza sativa ssp. japonica* variety *Dongbeixiang* with ^60^Co gamma irradiation, which showed high efficiency of Zn enrichment in grain (Wang et al. 2017). The *lpa* mutant *Os-lpa-XS110–1* was previously developed through ^60^Co gamma irradiation followed by NaN_3_ treatment of an *Oryza sativa ssp. japonica* variety *Xiushui110* (Liu et al. 2007). The *Os-lpa-XS110–1* has a pronounced reduction of phytic acid in seed compared to the original wild-type, which was attributed to the disruption of *OsMIK* by the insertion of a rearranged retrotransposon (Liu et al. 2007; Zhao et al. 2013). The double haploid (DH) progenies of different genotypes were developed from the *LLZ*×*Os-lpa-XS110–1* cross following a protocol adopted from Nguyen et al. (2016). In brief, *LLZ* was crossed with *Os-lpa-XS110–1* to generate F_1_ progenies. The anthers of F_1_ progenies were cultured on SK3 medium supplemented with 2,4-D (1.5 mg/L), casein hydrolysate (0.3 g/L) and sucrose (60 g/L), and phytagel (4 g/L) at pH 5.8 for callus induction in dark at 25 °C. After 25 days, calli were transferred to N6 medium supplemented with 6-BA (3 mg/L), casein hydrolysate (0.3 g/L), NAA (1 mg/L) and sucrose (30 g/L) at pH 5.8 for differentiation and regeneration. Regenerated plantlets were then transferred to 1/2 N6 medium supplemented with sucrose (20 g/L), colchicine (4 mg/L) and agar (7 g/L) at pH 5.8 under light. Plantlets with good root system were transplanted to paddy field..

Two rice mutant lines and their wild type parents were grown in the experimental field of Zhejiang Academy of Agricultural Sciences in Hangzhou (120°2′E, 30°3′N) during the rice-growing season of 2017 and 2018, and the DH progenies were grown in the same paddy field in 2018. For evaluating the environmental effects on Zn and phytic acid content, three rice lines for each type of cross-bred progenies were chosen based on similarity of flowering time, and grown on three sites in 2019, i.e. Fuyang (119°95′E, 30°05′N), Yuhang (120°3′E, 30°42′N) and Lin’an (119°72′E, 30°23′N). The respective soil types for each site (Yuhang, Fuyang and Lin’an) were fine silty clay, silty clay loam and fine sandy loam. In each site, each rice line was grown in a block containing 5 rows and 5 plants per row. After fully matured, seeds of the middle 9 plants (3 rows, 3 plants per row) were harvested, of which 3 individual plants were pooled and mixed completely. These seeds were dried in an oven at 50 °C until constant weight was obtained. For each sample, 30 dry seeds were hulled and used for subsequent analysis.

For hydroponic experiments, seeds were surface sterilized by 30% (v/v) sodium hypochlorite solution for 30 min, and washed several times with deionized water. Subsequently, the seeds were soaked in deionized water for 48 h at 30 °C and germinated for 6 days at 30 °C. Then the seedlings were transplanted into 48-well plastic buckets (35 L) with half-strength Kimura B solution (pH 5.6). After grown to the fifth leaf stage, the rice lines were treated with nutrient solution containing 0.4, 4 and 40 μmol/L Zn concentration respectively until maturity. The nutrient solution was renewed every 4 days, and the pH was kept at 5.5 by adjusting every day using 1 M NaOH or HCl. Hydroponic experiments were carried out in a greenhouse with a 13-h light/11-h dark photoperiod at 22–30 °C. All experiments were performed with three biological replicates.

### Mineral Analysis

The brown rice was dried at 75 °C for 48 h and grounded into powder. The samples were subjected to acid digestion in a closed-vessel microwave system using 5 mL nitric acid (Mars Express, CEM Corporation, Matthews, USA). After cooling, the digestion solution was transferred to a 25 mL volumetric flask, and the volume was added to 25 mL with 3% nitric acid. The concentrations of zinc (Zn), manganese (Mn), copper (Cu) and iron (Fe) in samples were determined by inductively coupled plasma mass spectrometry (ICP-MS, PlasmaQuant MS, Analytik Jena AG, Germany).

### Phytic Acid Determination

Phytic acid was extracted and determined according to Dai et al. (2007) with slight modification. The grounded rice flours of 0.5 g were weighed and placed into 50 mL polystyrene centrifuge tubes. The samples were extracted with 10 mL of 0.2 M HCl for 2 h, followed by centrifuged at 10000 g for 10 min. Supernatants of 2.5 mL was mixed with 2 ml of 0.2% FeCl_3_ solution and boiled in a water bath for 1 h. After centrifugation at 10000 g for 15 min, the precipitates were re-suspended with 5 mL deionized water and 3 mL 1.5 M NaOH by vortexing for 4 min. Afterwards, the precipitate was recollected by centrifugation at 10000 g for 15 min and washed three times with 5 mL of deionized water. The supernatant was discarded and 3 mL 0.5 M HCl was added to dissolve the residue. Finally, the volume of solutions was made up to 20 mL by deionized water. The iron concentration in the solution was measured by ICP-MS (PlasmaQuant MS, Analytik Jena AG, Germany). The phytic acid content was subsequently calculated by multiplying iron content by the factor 4.2.

### In Vitro Digestion

The in vitro digestion method was according to Wei et al. (2012b). Rice powder sample (0.5 g) was added with 0.5 mL of pepsin (0.2 g pepsin in 5 mL 0.1 M HCl, pH = 2.0) and incubated at 37 °C in a shaking water bath for 2 h. Afterwards, the mixtures were added with 2.5 mL pancreatin-bile solution (0.45 g bile salts and 0.075 g pancreatin in 37.5 mL 0.1 M NaHCO_3_, pH = 5.0) and then incubated at 37 °C in a shaking water bath for 2 h. The gastrointestinal digest was adjusted to pH 7.2, and boiled in a water bath for 4 min. The volume of the digest was brought to 15 ml with 120 mM NaCl and 5 mM KCl. After centrifugation at 3500 g for 1 h at 4 °C, the supernatant was transferred into a new 50 mL polystyrene centrifuge tube. Finally, glucose (5 mM the final concentration) and HEPES (50 mM final concentration) were respectively added into the solution, and the osmolarity was adjusted to 310 ± 10 mOsm/kg with deionized water. The solution was used for Zn uptake experiment in Caco-2 cell model.

### Zn Uptake Experiment in Caco-2 Cell Model

Caco-2 cells (passage 20) were obtained from the Institute of Biochemistry and Cell Biology, Chinese Academy of Sciences (Shanghai, China). The cells were cultured in high glucose (4.5 g/L) DMEM, which were supplemented with 10% (v/v) fetal bovine serum, 1% (v/v) antibiotic solution (penicillin−streptomycin), 1% (v/v) nonessential amino acids, 1% (v/v) L-glutamine, and 5 mM HEPES. The cells were maintained in an incubator with 5% CO_2_ and 95% relative humidity air at 37 °C. The medium was refreshed every 2 days. After reaching 80% confluence, cells were sub-cultured and digested by 0.25% trypsin-EDTA. When Caco-2 cells were between passages 30–46, they were seeded in a 6-well transwell plate (24 mm diameter, 0.4 μm pore size, Corning, USA) at density of 50,000 cells/cm^2^. Subsequently, 2.5 mL of complete DMEM was added into the basal chamber of transwell plate. The culture medium was changed every 2 day. After incubated for 21 days, the trans-epithelial electrical resistance (TEER) was measured for evaluating the cellular integrity of the monolayers. Caco-2 cell model with a TEER reading greater than 600 Ω cm^2^ could be used in Zn uptake experiment.

The growth medium was removed from each culture well, and the cell monolayer was washed three times with 37 °C HBSS. Afterwards, 2.5 mL of the transport solution (130 mM NaCl, 10 mM KCl, 1 mM MgSO_4_, 5 mM glucose, and 50 mM HEPES, pH 7.4) was added into the basal chamber of transwell plate, then the upper chamber was covered with 1.5 mL intestinal digestion solution. The cells were maintained in an incubator with 5% CO_2_ and 95% relative humidity air at 37 °C. After incubated for 2 h, the transport solution of the basal chamber was collected. The cell monolayers were harvested, followed by wash twice with ice cold HBSS. Then, 1 mL deionized water was added into the well, and the cells on filters were harvested after ultrasonic degradation. The Zn content of the gastrointestinal digestion solution, cell lysate, and the transport solution were analyzed by using an ICP-MS (PlasmaQuant MS, Analytik Jena AG, Germany). According to Wei et al. (2012b), the following equations were used to calculate the Zn bioavailability: Zn bioaccessibility (%) = Zn content in the solution after in vitro digestion (μg/g) × 100/total Zn content in brown rice (μg/g); Zn retention efficiency (%) = Zn retention (μg/well) × 100/Zn content in the solution after in vitro digestion (μg/well); Zn transport efficiency (%) = Zn transport (μg/well) × 100/Zn content in the digestion solution (μg/well); Zn uptake efficiency (%) = Zn retention efficiency + Zn transport efficiency; Bioavailable Zn content in the brown rice (μg/g) = Zn concentration in the brown rice (μg /g) × Zn bioaccessibility × Zn uptake efficiency /10000.

### Statistical Analysis

Data were analyzed using SPSS 24.0 (SPSS, IBM, Chicago, USA). For multiple comparisons, analysis of variance (ANOVA) was performed with the least significant difference (LSD) to compare the various means of each series of experiments when variances were homogenous. Means were considered to be significantly different if *P* values were ≤ 0.05. Pearson correlation analysis was carried out to analyze the correlation.

## Results

### Four Metallic Elements and Phytic Acid in the Brown Rice of Four Rice Lines Grown in Field Trial

Field experiments were conducted in 2017 and 2018. The yield-related agronomic traits of mutant lines and their wild-types were presented in Table S1. The seed setting rates of *LLZ* and *DBX* were higher than those of *Os-lpa-XS110–1* and *Xiushui110* (Table S1)*.* No significant consistent differences among four rice lines were observed for other agronomic traits (Table S1).

Phytic acid (PA) and four metallic elements, including Mn, Fe, Cu and Zn, were evaluated for each year (Tables 1 and 2). There was no significant difference in Mn content among four rice lines for each year (Table 1). The mutant *LLZ* and its wild-type parent *DBX* accumulated higher levels of Fe and Cu compared to *Os-lpa-XS110–1*and *Xiushui110* in each year, but the difference between varieties was small, even not significant for Fe content in 2018 (Table 1). There was a significant positive correlation with the contents of Zn and Fe or Cu, though the values of coefficient of determination (R^2^) were less than 50% (Table S2). It is noteworthy that the mutant *LLZ* exhibited the highest Zn density in brown rice, which was at least 1.88-fold higher than those of other three rice lines on average (Table 2).

**Table 1:** Contents of Fe, Mn and Cu in the brown rice of the mutant lines and their wild type parents

Year	Material	Mn (mg/kg)	Fe (mg/kg)	Cu (mg/kg)
2017	*LLZ*	23.26 ± 0.72a	14.29 ± 0.44a	6.37 ± 0.32a
	*DBX*	23.05 ± 0.97a	14.16 ± 0.42a	6.22 ± 0.39a
	*Os-lpa-XS110–1*	22.96 ± 0.90a	10.93 ± 0.39b	4.59 ± 0.29b
	*Xiushui110*	23.11 ± 0.74a	11.19 ± 0.48b	5.11 ± 0.23b
2018	*LLZ*	27.45 ± 0.90a	21.99 ± 0.92a	10.8 ± 0.41a
	*DBX*	27.83 ± 0.88a	20.94 ± 0.78ab	10.47 ± 0.43a
	*Os-lpa-XS110–1*	25.92 ± 1.00a	18.59 ± 0.75b	7.04 ± 0.47b
	*Xiushui110*	26.71 ± 1.08a	18.95 ± 0.74ab	8.85 ± 0.74ab

Data represent the mean ± SEM of 4 samples replicate. Different letters behind the values indicate statistically significant differences by LSD (*P* < 0.05).

**Table 2:** Zn, phytic acid and their molar ratio in the brown rice of the mutant lines and their wild type parents

Year	Material	Zn (mg/kg)	PA (mg/g)	[PA]/[Zn] (molar ratio)
2017	*LLZ*	46.65 ± 0.80a	7.39 ± 0.08a	15.62 ± 0.21c
	*DBX*	25.55 ± 0.99b	7.12 ± 0.06b	27.58 ± 1.27b
	*Os-lpa-XS110–1*	20.81 ± 0.69c	3.65 ± 0.07d	17.33 ± 0.41c
	*Xiushui110*	21.56 ± 0.56c	6.83 ± 0.07c	31.3 ± 1.04a
2018	*LLZ*	52.91 ± 1.23a	7.61 ± 0.04a	13.61 ± 0.36c
	*DBX*	27.34 ± 0.98b	7.27 ± 0.09b	26.51 ± 0.64a
	*Os-lpa-XS110–1*	22.17 ± 0.96c	4.09 ± 0.08c	17.11 ± 0.60b
	*Xiushui110*	23.52 ± 0.82c	7.02 ± 0.06b	28.67 ± 1.85a

Data represent the mean ± SEM of 4 samples replicate. Different letters behind the values indicate statistically significant differences by LSD (*P* < 0.05).

The phytic acid content in brown rice varied among four rice lines. The mutant *LLZ* had the highest phytic acid content in brown rice, followed by *DBX* and *Xiushui110* (Table 2). The *lpa* mutant *Os-lpa-XS110–1* exhibited the lowest phytic acid contents consistently in two field trials, and the average phytic acid contents in brown rice of *Os-lpa-XS110–1* were 48.4% lower than that in *LLZ* (Table 2).

The molar ratios of phytic acid to Zn in the brown rice ranged from 13.44 to 16.15 in *LLZ*, 23.48 to 30.77 in *DBX*, 16.11 to 20.32 in *Os-lpa-XS110–1*, and 27.70 to 33.91 in *Xiushui110* (Table 2). Two mutant lines displayed lower molar ratio of phytic acid to Zn in brown rice.

### Comparison of Zn and Phytic Acid Contents between *LLZ* and *Os-lpa-XS110–1* in Response to Zn Treatment

For evaluating the effects of Zn supply on the Zn and phytic acid content, the *LLZ* and *Os-lpa-XS110–1* were grown in nutrient solution with three Zn concentrations (0.4, 4 and 40 μM). Both rice lines appeared to be affected by elevated Zn supply, resulting in significant reduction of biomass, plant height, root length and seed-setting (Table S3). However, grain weight was not significantly affected by high Zn stress in both rice lines (Table S3).

**Fig. 1:**
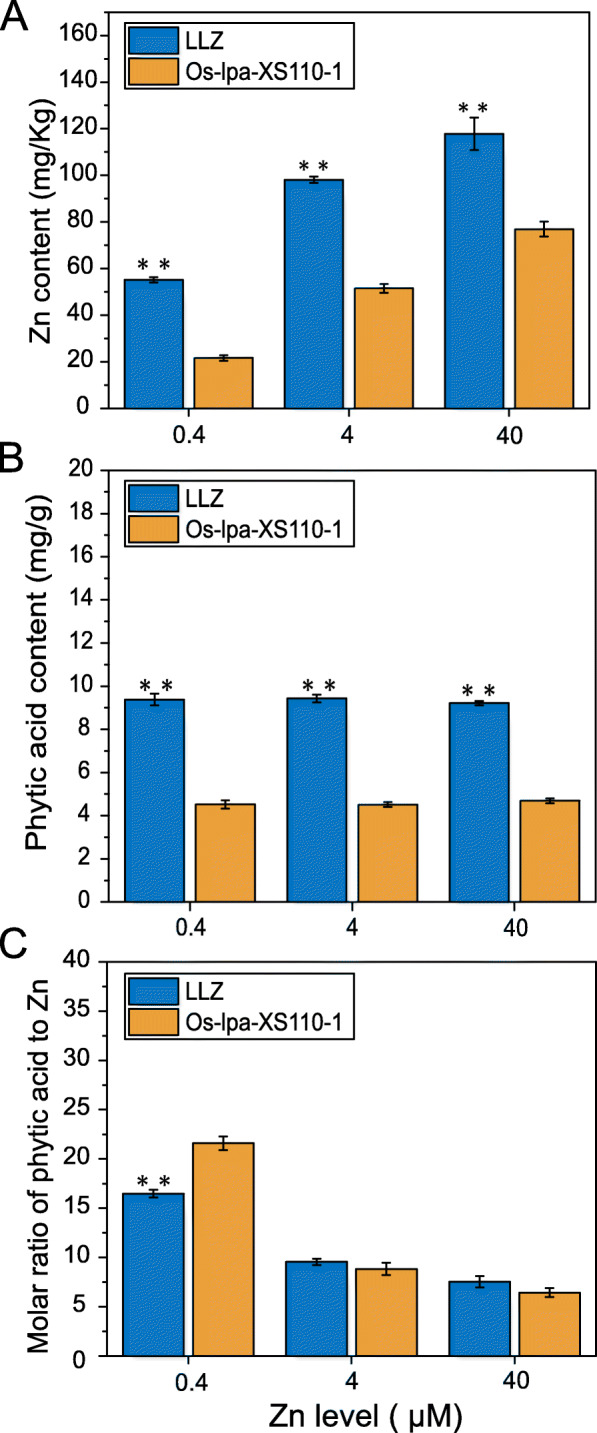
Contents of Zn and phytic acid in *LLZ* and *Os-lpa-XS110–1* under different Zn conditions. **a** Zn content in the brown rice; **b** Phytic acid content in the brown rice; **c** Molar ratio of phytic acid to Zn in the brown rice. The results are presented as mean ± SEM. Asterisks represent statistically significant differences between *LLZ* and *Os-lpa-XS110–1* (*n* = 4; *P < 0.05; **P < 0.01)

Mineral analysis showed that additional Zn supply had a large effect on grain Zn content in *LLZ* and *Os-lpa-XS110–1*. With elevated Zn supply, the Zn content in brown rice increased from 55.15 to 117.79 mg/kg in *LLZ*, but only 21.63 to 76.92 mg/kg in *Os-lpa-XS110–1* (Fig. 1a). The average Zn content in *LLZ* were 2.55-fold, 1.90-fold and 1.53-fold, respectively, higher than those in *Os-lpa-XS110–1* under 0.4, 4 and 40 μM Zn conditions (Fig. 1a). While the phytic acid content was not significantly affected by extra Zn supply, the *LLZ* had significantly higher phytic acid content in brown rice compared to *Os-lpa-XS110–1* under each Zn condition, and the average phytic acid content of *LLZ* was at least 2.55-fold than that in *Os-lpa-XS110–1* (Fig. 1b)*.* Consequently, the molar ratios of phytic acid to Zn showed a negative response to increasing Zn supply (Fig. 1c). The molar ratios of phytic acid to Zn in *LLZ* was significantly lower than that in *Os-lpa-XS110–1* when exposed to 0.4 μM Zn treatment*,* whereas it did not significantly differ between two mutant lines under 4 and 40 μM Zn conditions (Fig. 1c).

### Contents of Zn and Phytic Acid in Cross-Bred Progenies

We obtained a total of 120 DH homozygous progenies through anther culture. The Zn contents in brown rice of homozygous progenies varied from 13.12 mg/kg to 55.16 mg/kg with the mean value of 33.94 mg/kg, while the phytic acid content varied from 2.14 mg/g to 8.45 mg/g with the mean value of 5.45 mg/g (Table 3). The Zn content in progeny was not significantly correlated to the phytic acid content (*r* = 0.207, *P*<0.05).

**Table 3:** Zn, phytic acid and their molar ratio in the brown rice of cross-bred progenies

Progeny	Number of lines	Zn (mg/kg)		PA (mg/g)		[PA]/[Zn] (molar ratio)	
		Mean	CV	Mean	CV	Mean	CV
Total	120	33.94	0.36	5.45	0.33	17.75	0.46
Wild-type	24	21.48c	0.17	6.88a	0.13	31.87a	0.08
LLZ-type	35	42.63b	0.13	7.24a	0.09	16.92b	0.12
Lpa-type	29	21.23c	0.19	3.62b	0.22	16.82b	0.13
DM-type	32	45.30a	0.19	4.06b	0.13	8.89c	0.12

Different letters behind the values indicate statistically significant differences by LSD (*P* < 0.05)

**Fig. 2:**
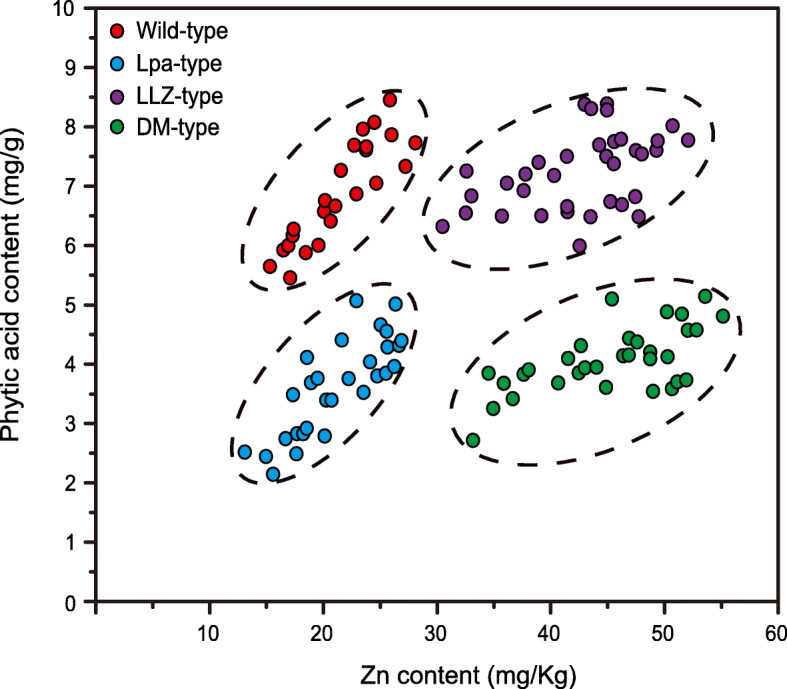
Distribution of Zn and phytic acid content in brown rice for the cross-bred progenies

Based on the Zn and phytic acid content in brown rice, the DH progenies could be classified into four types, including wild-type, LLZ-type, Lpa-type and double-mutant-type (DM-type in short) (Fig. 2). The χ^2^ goodness-of-fit test indicated that the segregation ratio of these progenies fitted a 1:1:1:1 ratio (χ^2^ = 2.2, *P* = 0.532). The wild-type progenies showed a similar phenotype to *Xiushui110*, with the average 21.48 mg/kg Zn and 6.88 mg/g phytic acid in the brown rice (Table 3). The LLZ-type progenies had a higher Zn level in the brown rice, around 2-fold higher than those in the wild-type, whereas it also had the highest phytic acid content (7.24 mg/g) among all four types (Table 3). In contrast to LLZ-type progenies, the Lpa-type progenies exhibited a significantly decreased level of phytic acid (3.62 mg/g) compared to the wild-type, but it had only 21.23 mg/kg Zn in the brown rice (Table 3). The DM-type progenies displayed the highest level of Zn (45.30 mg/kg) and lower level of phytic acid (4.06 mg/g) in the brown rice (Table 3). Thus, the molar ratio of phytic acid to Zn of the DM-type was lowest, which was a quarter of that of the wild-type progeny (Table 3). What’s more, the contents of other three metallic elements (Mn, Fe and Cu) exhibited similar among four types of progenies (Table S4).

### Environmental Impact on the Zn and Phytic Acid Content in Cross-Bred Progenies

Three rice lines for each type of progenies were selected for field trials at three sites (Yuhang, Fuyang and Lin’an). The yield-related agronomic traits for these progenies are presented in Figure S1. Due to significantly greater number of grains per plant in Yuhang, LLZ-type in Yuhang had the significant higher yield compared to that in Fuyang and Lin’an (Figure S1). The other types of progenies from three field trials exhibited the same pattern regarding the agronomic traits (Figure S1).

**Fig. 3:**
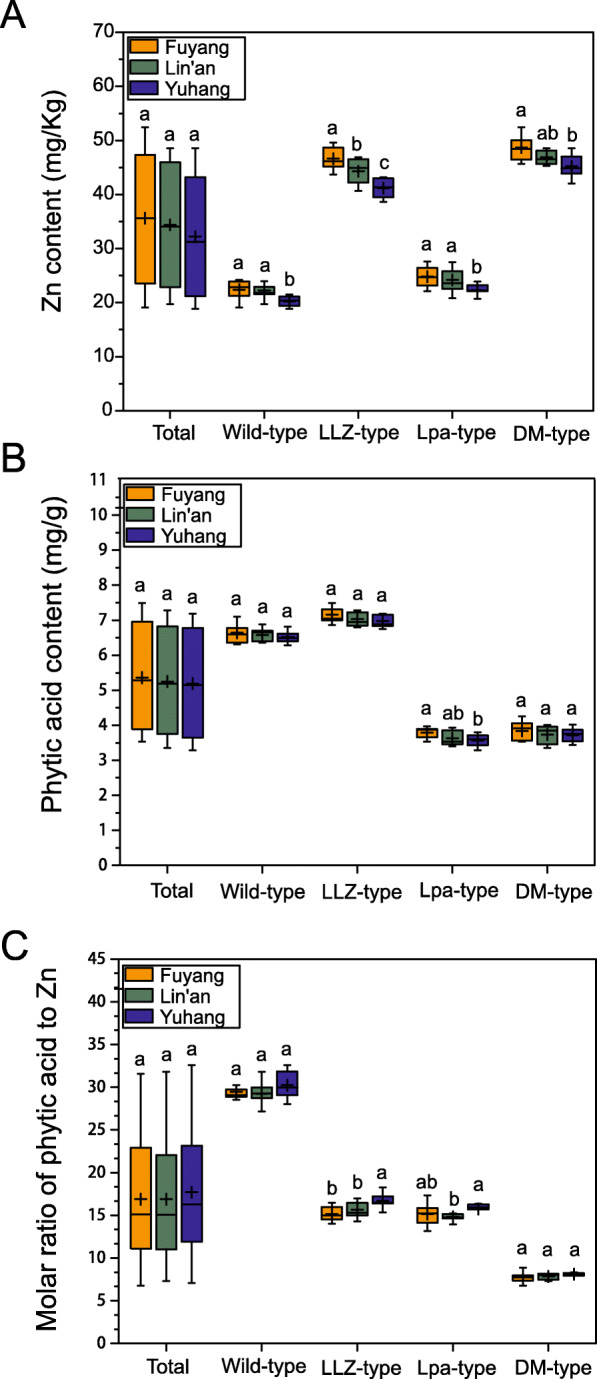
Environmental impact on Zn and phytic acid contents in cross-bred progenies. Three rice lines for each type of progenies were planted at three sites (Yuhang, Fuyang and Lin’an). **a** Zn content in the brown rice. **b** Phytic acid content in the brown rice. **c** Molar ratio of phytic acid to Zn in the brown rice. The results are presented as box-and-whisker plots. Plus symbol: mean value; line in the box: median value; bottom and top of the box: first quartile (Q1) and third quartile (Q3); upper whisker: top of box + 1.5 × interquartile range (IQR = Q3 - Q1); lower whisker: bottom of box - 1.5 × IQR. Different letters indicate statistically significant differences by LSD (*P* < 0.05)

There were no statistically significant differences in the average contents of Zn and phytic acid for all hybrid progenies among three sites (Fig. 3a and b). All four types grown in Yuhang had significantly lower Zn contents than those grown in other sites; LLZ-type and DM-type grown in Fuyang had the higher Zn contents, but the Zn content in Lpa-type and wild-type grown in Fuyang and Lin’an did not differ significantly (Fig. 3a). In contrast, the phytic acid contents in each type showed similar levels in three sites except Lpa-type, which had the significantly higher content of phytic acid grown in Fuyang than that in Yuhang (Fig. 3b). For each field trial, the molar ratio of phytic acid to Zn was lowest in the DM-type progeny, intermediate in the LLZ-type and Lpa-type, and the highest in the wild-type progeny (Fig. 3c).

### Zn Bioavailability in Cross-Bred Progenies

The cross-bred progenies were used to assess Zn bioavailability through in vitro digestion/ Caco-2 cell culture model. The LLZ-type and DM-type showed the higher Zn bioaccessibility (30.47% and 28.34%, respectively), whereas the wild-type and Lpa-type progenies had only 20.80% and 24.13% Zn bioaccessibility respectively (Table 4). In contrast, the Lpa-type had the highest Zn uptake efficiency (30.48%), followed by DM-type (23.05%) and LLZ-type (17.01%), with the wild-type progeny (13.58%) having the lowest percentages (Table 4). There was a significant correlation between total Zn content and the bioaccessible Zn content (*r* = 0.727, *P* = 0.007).

**Table 4:** Zn bioavailability in the brown rice of cross-bred progenies

Progeny	Bioaccessibility (%)	Efficiency (%)			Bioavailable Zn (μg/g grain)
		Retention	Transport	Zn uptake	
LLZ-type	30.47 ± 1.01a	6.55 ± 0.78c	10.45 ± 0.37c	17.01 ± 0. 82c	2.29 ± 0.13b
Lpa-type	24.13 ± 1.37b	13.78 ± 0.47a	16.70 ± 0.49a	30.48 ± 1.76a	1.58 ± 0.05c
DM-type	28.34 ± 1.18a	10.16 ± 1.23b	12.89 ± 0.44b	23.05 ± 1.22b	3.21 ± 0.14a
Wild-type	20.80 ± 0.88b	5.15 ± 1.04c	8.42 ± 0.53d	13.58 ± 1.21d	0.67 ± 0.06d

Data represent the mean ± SEM of 4 samples replicate. Different letters behind the values indicate statistically significant differences by LSD (*P* < 0.05).

There was a significant difference in the bioavailable Zn content among four types. The average content of bioavailable Zn was 0.67 μg/g in wild-type, 2.29 μg/g in LLZ-type, 1.58 μg/g in Lpa-type and 3.21 μg/g in DM-type (Table 4). The level of bioavailable Zn was significantly correlated with Zn bioaccessibility (*r* = 0.780, *P* = 0.003) and Zn uptake efficiency (*r* = 0.627, *P* = 0.029). Due to the relatively high Zn bioaccessibility and Zn uptake efficiency, DM-type contained the highest content of bioavailable Zn in brown rice (Table 4). The average amount of bioavailable Zn in the DM-type progeny was 4.78-fold greater than that in wild-type progeny (Table 4).

## Discussion

In this study, two previously developed rice mutants were selected as the parents to generate hybrid progenies. The mutant *LLZ* exhibited a high Zn density in grain, while the *Os-lpa-XS110–1* accumulated significantly lower level of phytic acid. Phytic acid is the principal storage form of phosphorus (P) in rice grain, which will chelate with Zn to form a complex (O'Dell et al. 1972; Iwai et al. 2012). The negative relationship between phytic acid and Zn had been observed in previous studies, in which the decreased phytic acid in *lpa* mutants would increase grain Zn content (Karmakar et al. 2020; Liu et al. 2004). However, Sakai et al. (2015) concluded that the phytic acid content did not affect the Zn accumulation in rice seed. Yatou et al. (2018) found that there was not an obvious correlation between phytic acid content and Zn content. In our study, the cross-bred progenies exhibited diverse contents of Zn and phytic acid in grain (Fig. 2). This suggested that phytic acid biosynthesis and Zn loading into grain are controlled by different regulatory mechanisms, and it is potential for manipulating the phytic acid content without affecting the Zn accumulation.

Genotypic differences in the contents of phytic acid and Zn became significant between two hybrid parents, independent of environmental conditions (Table 2). A similar pattern of environmental impact has been determined for hybrid progenies of *LLZ*/*Os-pla-XS110* grown on diverse sites (Fig. 3). Previous studies indicated that Zn and phytic acid in rice grain were complex traits with the involvement of multiple genes, and were significantly influenced by environments (Ahn et al. 2010; Wissuwa et al. 2008). Several rice populations have also been used to assess the variability for Zn and phytic acid content in grain, suggesting that the variances of Zn and phytic acid contents were attributed to genotypes, environments and interactions between the two factors (Garcia-Nebot et al. 2013; Norton et al. 2014; Zhang et al. 2008b). However, we found that the genotype was by far the dominant factor to determine Zn and phytic acid content in brown rice, and there was no significant interaction between genotype and year on the Zn and phytic acid content (Table S5).

Due to the variety-specific distribution of Zn in rice, the effect of additional Zn supply on grain Zn content depended on rice genotypes and Zn conditions. Gao et al. (2006) showed that improved Zn nutrition significantly enhanced grain Zn concentration in either paddy rice or aerobic rice under aerobic and flooded cultivations. Wissuwa et al. (2008) concluded that the Zn supply had a significant positive effect on grain Zn concentrations in the upland soil, whereas application of Zn supply to Zn deficient soil rendered a concomitant decrease in gain Zn content. The Zn contents in the brown rice increased by 0.8-fold in *LLZ* and 1.4-fold in *Os-lpa-XS110–1* from 0.4 to 4 μM Zn conditions respectively but only increased by 0.2-fold in *LLZ* and 0.5-fold in *Os-lpa-XS110–1* from 4 to 40 μM Zn conditions (Fig. 1a). Zn in the grain is thought to be supplied as complexes with ligands via the phloem after mobilization from vegetable tissues, and the synthesis of some Zn-binding ligands was becoming saturated with consistently elevated Zn supply, which was the main limiting factor to determine grain Zn content under Zn-sufficient conditions (Nishiyama et al. 2013; Wang et al. 2017; Wu et al. 2010). Thus, the magnitude of grain Zn enrichment descended with elevated Zn supply. In contrast, the grain phytic acid content was not significantly affected by high Zn stress (Fig. 1b). This result was inconsistent with previous study (Wei et al. 2012b), in which application of Zn fortification during the germination process could significantly reduce phytic acid content in brown rice. However, germination is a complex process during which the seed would quickly activate enzymes to hydrolyze phytic acid for providing nutrient requirement (Bollmann et al. 1980). Zn is an important cofactor for enzymes, and exogenous Zn supply will increase the grain Zn content to accelerate the hydrolysis of phytic acid. Nevertheless, our present results further indicated that phytic acid, a major storage form of phosphorus in rice grain, has different homeostasis mechanism from Zn. It is noteworthy that high Zn supply inhibited the growth of *LLZ* and *Os-lpa-XS110–1*, exhibited as decreased dry weight, plant height and root length (Table S3), and affected the grain production system of rice as the seeding-setting was significantly decreased although the grain weight seemed unaffected (Table S3). Similar results also found in previous reports (Impa et al. 2013; Jiang et al. 2007; Wang et al. 2017).

Many studies found that there were significant correlations between Zn and other metallic elements in rice grain (Della Valle and Glahn 2014; Hao et al. 2007; Zhang et al. 2018; Zhang et al. 2008a). Several high Zn density rice varieties would concomitantly increase the contents of other metallic elements in grains. Graham et al. (1999) found two high Zn density rice varieties would concomitantly increase the grain Fe contents. Two high Fe density mutant M-IR-75 and M-IR-58 were identified from the rice variety IR64, which had a common level of Zn in the grain (Jeng et al. 2012). Among the 274 rice varieties analyzed, Jiang et al. (2007) proposed that Zn content had closely associations with other three metallic element contents (Fe, Cu and Mn) in milled rice. We found the mutant *LLZ* also accumulated considerably higher levels of Cu and Fe in grain compared to the *Os-pla-XS110–1* (Table 1), and Zn content showed a significant positive correlation with the contents of Fe and Cu (Table S2). While LLZ-type and DM-type progenies accumulated significantly more Zn contents in grains compared to Lpa-type and wild-type progenies (Table 3) but the Mn, Fe and Cu content in four hybrid progenies types were similar (Table S4). That indicated the great accumulation of Zn in grain may result in a significant increase in the contents of Fe and Cu but with a limited extent.

As assessed in Caco-2 cell model following in vitro digestion, Zn bioavailability of rice grain is dependent on Zn bioaccessibility and Zn uptake efficiency (Table 4). Zn bioaccessibility refers to the fraction of soluble Zn in the digestion that was released from the rice grain. Zn bioaccessibility has been shown to be affected by Zn speciation, the digestion phase and various dietary components (Zhang et al. 2020). The higher amount of bioaccessible Zn in progenies with high Zn density indicated that total Zn content had a positive effect on Zn bioaccessibility (Table 4). Wei et al. (2012b) also found the amount of soluble Zn in rice grain was increased by Zn fortification, and the total Zn content is the important factor to determine Zn bioaccessibility.

Zn uptake efficiency reflected the absorptive capacity of Zn in the Caco-2 cells, which was affected by dietary factors such as phytic acid, amino acids and other low-molecular weight ions (Lonnerdal 2000). Phytic acid in rice was the primary inhibitor to reduce Zn absorption by complexing with Zn in the human body (Hambidge et al. 2011; Raboy 2009). Several studies confirmed that the addition of phytic acid to the diet significantly lower the Zn absorption in human body (Hambidge et al. 2011; Lonnerdal et al. 1984; Miller et al. 2007). Sreenivasulu et al. (2008) found that Zn uptake was inhibited by phytic acid in Caco-2 cells, and phytic acid content was negatively correlated with Zn bioavailability. Similar results were obtained in our study, in which the Lpa-type and DM-type progenies had the higher percentage of Zn retention, transport, and uptake efficiency by Caco-2 cell than other type progenies (Table 4). Zn uptake efficiency was significantly negatively correlated with the phytic acid content in brown rice (*r* = 0.665, *P* = 0.018). However, Jou et al. (2012) reported that the elevated content of phytic acid did not affect Zn absorption in the biofortified rice, which may be caused by the unchanged molar ratio of phytic acid to Zn.

The molar ratio of phytic acid to Zn seems to be an effective evaluation parameter for Zn bioavailability. According to World Health Organization, less than 15% Zn from food is absorbed when the molar ratio of phytic acid to Zn was greater than 15 (Allen et al. 2006). Fredlund et al. (2006) studied the dose-dependent inhibitory effects of phytic acid on Zn absorption in man, and found that the zinc absorption was significantly decreased when the molar ratio of phytic acid to Zn in the meals increased from 2.9 to 11.5. Jou et al. (2012) exhibited a similar result that a dose-responsive decrease in Zn uptake could be observed in either Caco-2 cells or rat pups when the molar ratio of phytic acid to Zn molar increased from 2.5 to 20. Lonnerdal et al. (2011) found that the molar ratio of phytic acid to Zn was lower in the low phytic acid mutants than that in the WT, leading to substantial increases in Zn absorption in the low phytic acid mutants. That indicated it is feasible to improve Zn bioavailability by increasing Zn contents and reducing phytic acid contents in rice grain. Indeed, double mutant obtained by pyramiding breeding showed a lower molar ratio of phytic acid to Zn and higher Zn bioavailability than either low phytic acid mutant or high Zn mutant (Tables 3, 4). The double mutant seems to be a valuable genetic material that could be applicable to Zn biofortification breeding. However, the in vitro digestion/Caco-2 cell model is a preliminary method to evaluate Zn bioavailability. To predict the effect of cross-breeding on the nutritional status of individuals, these progenies will be further assessed in human feeding trials.

## Conclusions

The present study shows a practical way to generate a double mutant with high Zn bioavailability by crossing high Zn density mutant and low phytic acid mutant. The double mutant progeny exhibited significant increment in Zn content and reduction in phytic acid content in brown rice. The results from the in vitro digestion/Caco-2 cell model indicated the double mutant progeny had a higher Zn bioavailability than other progenies. These demonstrate that pyramiding breeding is an effective method to improve the amount of Zn and Zn bioavailability in rice grain.

## Supplementary Information

**Additional file 1: Figure S1**.The yield-related agronomic traits of cross-bred progenies grown in different field trials.

**Additional file 2: Table S1**. Comparison of yields and yield-related traits between the mutant lines and their wild type parents. **Table S2**. Relationship of four metallic elements in four rice lines. **Table S3**. Differences itn plant growth between *LLZ* and *Os-lpa-XS110–1* under different Zn conditions. **Table S4**. Contents of Mn, Fe and Cu in the brown rice of cross-bred progenies. **Table S5**. ANOVA for the phytic acid and Zn content in brown rice.

## Data Availability

The datasets supporting the conclusions of this article are included within the article and its additional files.

## Abbreviations

ANOVA: Analysis of variance
CV: Coefficient of variation
DH: Double haploid
DMEM: Dulbecco’s modified Eagle medium
DM-type: Double-mutant-type
FBS: Fetal bovine serum
HBSS: Hanks’ balanced salt solution
HEPES: L-glutamine and 4-(2-hydroxyethyl)-1-piperazineethanesulfonic acid
ICP-MS: Inductively coupled plasma mass spectrometry
lpa: Low phytic acid
LSD: Least significant difference
PA: Phytic acid
TEER: Trans-epithelial electrical resistance
