# Supplementary material for: Combination of High Zn Density and Low Phytic Acid for Improving Zn Bioavailability in Rice (Oryza stavia L.) Grain
Source: Rice (N Y). 2021 Feb 27;14:23. doi: 10.1186/s12284-021-00465-0 (PMC7914331; doi:10.1186/s12284-021-00465-0)
Supplement: Supplementary file 1 — Additional file 1: Figure S1.The yield-related agronomic traits of cross-bred progenies grown in different field trials. [file 12284_2021_465_MOESM1_ESM.docx]

**Supplementary information**

Combination of high Zn density and low phytic acid for improving Zn bioavailability in rice (*Oryza stavia* L.) grain

Yin Wang ^*,1,2^, Yusha Meng ^1,2^, Yanping Ma ^1,2^, Lei Liu ^1,2^, Dianxing Wu ^3^, Xiaoli Shu ^3^, Liqing Pan ^4^, Qixian Lai ^1,2^

1 Institute of Rural Development, Zhejiang Academy of Agricultural Sciences, Hangzhou 310021, China

2 Key Laboratory of Creative Agriculture, Ministry of Agriculture, Hangzhou 310021, China

3 State Key Laboratory of Rice Biology, Institute of Nuclear Agriculture Sciences, Zhejiang University, Hangzhou 310029, China

4 Yuyao County Agricultural Techniques Promotion and Service Station, Yuyao Agricultural and Rural Bureau, Ningbo 315400, China

* Corresponding author

Email: [wangyin@zaas.ac.cn](mailto:wangyin@zaas.ac.cn)

Figure Legends

**Fig.S1.** The yield-related agronomic traits of cross-bred progenies grown in different field trials. (A) Total grain number per plant; (B) Seed setting; (C) Yield per plant; (D) 1000 grain weight. The results are presented as box-and-whisker plots. Plus symbol: mean value; line in the box: median value; bottom and top of the box: first quartile (Q1) and third quartile (Q3); upper whisker: top of box + 1.5 × interquartile range (IQR = Q3 - Q1); lower whisker: bottom of box - 1.5 × IQR. Different letters indicate statistically significant differences by LSD (n=9; P < 0.05).


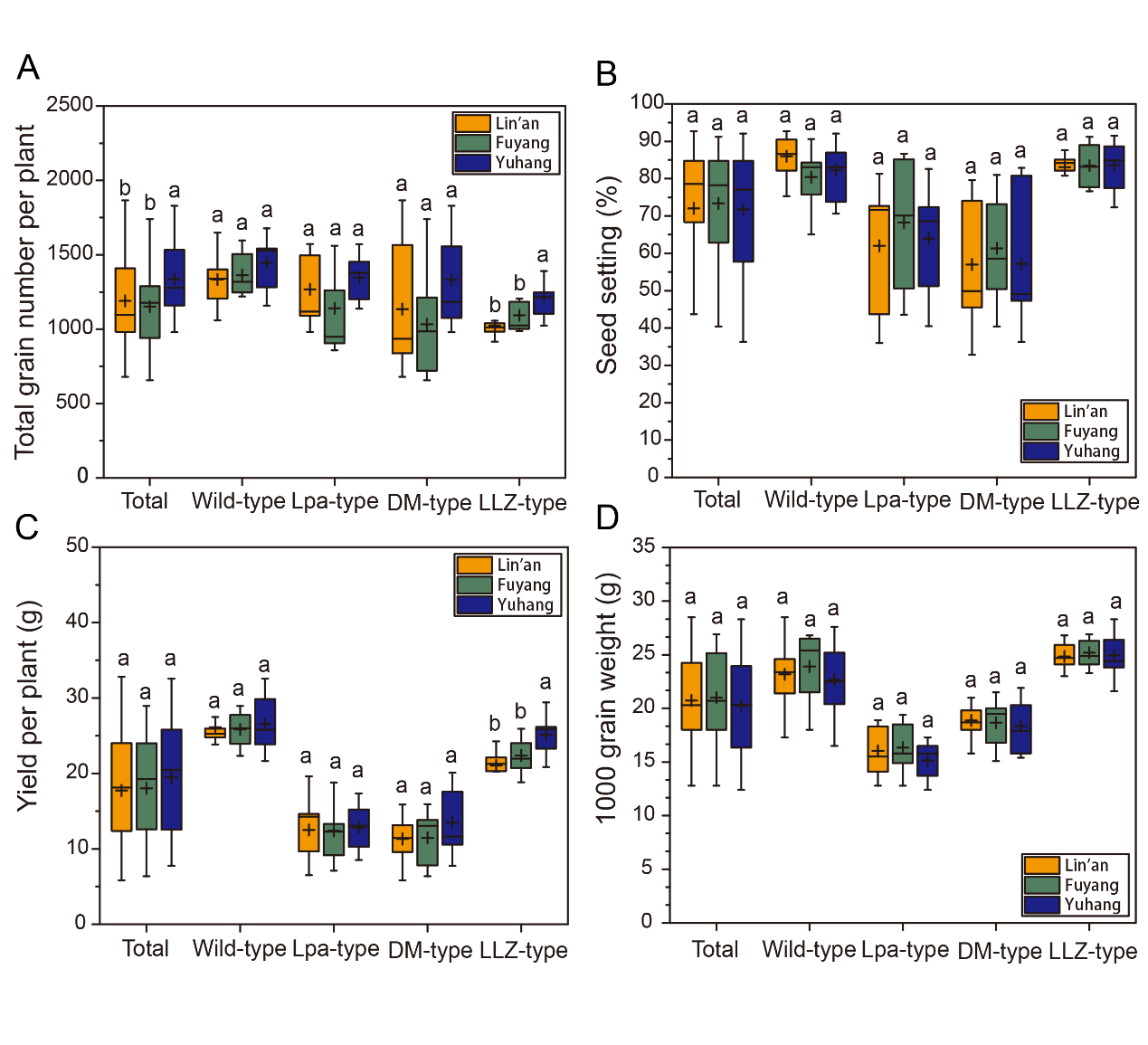


Fig.S1
